# A TTFS-based energy and utilization efficient neuromorphic CNN accelerator

**DOI:** 10.3389/fnins.2023.1121592

**Published:** 2023-05-05

**Authors:** Miao Yu, Tingting Xiang, Srivatsa P., Kyle Timothy Ng Chu, Burin Amornpaisannon, Yaswanth Tavva, Venkata Pavan Kumar Miriyala, Trevor E. Carlson

**Affiliations:** ^1^School of Computing, Department of Computer Science, National University of Singapore, Singapore, Singapore; ^2^School of Interactive Computing, Georgia Institute of Technology, Atlanta, GA, United States; ^3^Centre for Quantum Technologies, National University of Singapore, Singapore, Singapore

**Keywords:** artificial neural networks (ANNs), brain-inspired networks, neuromorphic hardware, spiking neural networks (SNNs), time-to-first-spike

## Abstract

Spiking neural networks (SNNs), which are a form of neuromorphic, brain-inspired AI, have the potential to be a power-efficient alternative to artificial neural networks (ANNs). Spikes that occur in SNN systems, also known as activations, tend to be extremely sparse, and low in number. This minimizes the number of data accesses typically needed for processing. In addition, SNN systems are typically designed to use addition operations which consume much less energy than the typical multiply and accumulate operations used in DNN systems. The vast majority of neuromorphic hardware designs support rate-based SNNs, where the information is encoded by spike rates. Generally, rate-based SNNs can be inefficient as a large number of spikes will be transmitted and processed during inference. One coding scheme that has the potential to improve efficiency is the time-to-first-spike (TTFS) coding, where the information isn't presented through the frequency of spikes, but instead through the relative spike arrival time. In TTFS-based SNNs, each neuron can only spike once during the entire inference process, and this results in high sparsity. The activation sparsity of TTFS-based SNNs is higher than rate-based SNNs, but TTFS-based SNNs have yet to achieve the same accuracy as rate-based SNNs. In this work, we propose two key improvements for TTFS-based SNN systems: (1) a novel optimization algorithm to improve the accuracy of TTFS-based SNNs and (2) a novel hardware accelerator for TTFS-based SNNs that uses a scalable and low-power design. Our work in TTFS coding and training improves the accuracy of TTFS-based SNNs to achieve state-of-the-art results on the MNIST and Fashion-MNIST datasets. Meanwhile, our work reduces the power consumption by at least 2.4×, 25.9×, and 38.4× over the state-of-the-art neuromorphic hardware on MNIST, Fashion-MNIST, and CIFAR10, respectively.

## 1. Introduction

Artificial neural networks (ANNs) have emerged as the most promising candidates for performing a wide range of tasks such as image classification and recognition, object detection, speech recognition, and speech-to-text translation. There have been significant improvements in the classification accuracies of ANNs, and in 2015, ANNs achieved human-level accuracy (He et al., 2015) at the ImageNet 2012 Visual Recognition Challenge. However, the high classification performance of ANNs comes at the expense of a large number of memory accesses and compute operations, which results in higher power and energy consumption. Recently, there has been an increased focus on developing more efficient ANNs (Howard et al., 2019; Tan and Le, 2019).

While these efficient networks provide a promising pathway toward the deployment of artificial intelligence (AI) in low-power devices, the use of spiking neural networks (SNNs) improves power savings even more. First, data are represented in binary, so the computation is done by only addition. Furthermore, addition operations are only performed when input is received, whereas ANN performs multiplications on all neurons across all inputs. In SNNs, information is represented and transmitted in the form of binary events called spikes, i.e., similar to the way information is encoded and propagated in the human brain. To process the information encoded, SNNs only require addition operations, whereas the standard ANNs require computationally expensive multiply-and-accumulate (MAC) operations. Furthermore, sparse activations (Khoei et al., 2020) lead to a significant reduction in the data movement between memory and processing elements. For these reasons, SNNs have garnered interest as potential candidates for designing the next generation of low-power AI accelerators (Bouvier et al., 2019).

There are a few prominent fully digital SNN accelerators such as SpiNNaker (Khan et al., 2008), TrueNorth (Merolla et al., 2014), and Loihi (Davies et al., 2018). SpiNNaker (Khan et al., 2008) consists of ARM processors interconnected through a custom Network-on-Chip (NoC). SpiNNaker is highly reconfigurable that can provide flexibility but has lower energy efficiency and speedup when accelerating complex neuron models and synapses as it is based on traditional CPUs. TrueNorth (Merolla et al., 2014) is a fully functional ASIC chip with 1 million spiking neurons and 256 million synapses. TrueNorth was innovative in many ways, including providing a very low-power density of just 20 milliwatts per square centimeter and supporting high-spike rates, which is the number of spikes fired within a time window. Unfortunately, they only support a limited number of neuron models and limited bit-precision for weights (Bouvier et al., 2019), and do not take into account activation sparsity, which is the ratio of non-zero values to the total number of activations. On the other hand, Loihi (Davies et al., 2018) is a fully asynchronous neuromorphic chip that can take advantage of the sparse activations present in SNNs, support many complex neuron models, and facilitate on-chip training of SNNs with different spiking-time-dependent-plasticity rules. Furthermore, a comparison of Loihi to general-purpose CPUs and GPUs in SNN acceleration has shown a 1, 000× efficiency improvement (Mayberry, 2017). While a significant efficiency improvement, SNN accelerators using rate coding continue to suffer from higher levels of energy consumption (For a detailed comparison of coding method efficiencies, see Section 2.1).

As an alternative to the higher power-consuming rate coding schemes, some works have looked at temporal coding (Rueckauer and Liu, 2018; Comsa et al., 2020) as a solution. One such type of temporal coding is time-to-first-spike (TTFS) coding (Rueckauer and Liu, 2018). In TTFS coding, the relative time of arrival of the spikes with respect to the appearance of the first spike represents the information, not the average number of spikes over a time period. Consequently, the number of spikes being generated and processed in the network is reduced significantly. This drop in spiking activity can lead to reductions in the number of memory accesses, total accelerator power, and energy consumption. However, in accelerators like TrueNorth, cores always require memory access for each neuron on each time step irrespective of spiking activity. As a result, the drop in spiking activity does not proportionally reduce the total power and energy consumption, making TrueNorth unsuitable for accelerating the TTFS-based SNNs.

To achieve better performance with temporal coding, a recent design, SpinalFlow (Narayanan et al., 2020), proposes a new structure and a new dimension for reuse patterns. It adopts an ordering of computations such that the outputs of a network layer are also compressed, time-stamped, and sorted. All relevant computations for a neuron are performed in consecutive steps to eliminate neuron potential storage overheads. While SpinalFlow can achieve better energy efficiency with better data reuse, it suffers from several drawbacks: (1) inefficient dataflow, (2) low PE utilization, and (3) cannot support max-pooling layer. Meanwhile, SpinalFlow requires large buffers to store and re-order input. More detailed analyses are given in Section 2.2.2.

With respect to dataflow improvements [drawback (1) above], we show that for highly sparse neural networks, especially for the TTFS-based SNNs, the use of the input stationary dataflow pattern can be more power efficient than the output stationary pattern (see Section 2.2 for details). We, therefore, focus on input stationary dataflow patterns in this work. To achieve a high PE utilization, we propose a mapping algorithm that can fully utilize all PEs when there is a spike required to be processed. Different from SpinalFlow which maps each neuron of one spine (their data structure) to each PE, this work maps the whole single output feature map on one PE. Whenever one input spike comes to the PEs, all PEs will be active. This mapping algorithm, as we propose in this work, leads a PE utilization near 100%. From the hardware perspective, our work can support different types of layers such as max-pooling layers, convolutional layers, and fully connected layers. Meanwhile, this work treats each input spike as an event, which means there is no input buffer or buffer re-ordering is needed in our design, which can be a time- and power-consuming step in SpinalFlow.

In addition to the power efficiency of our hardware, we also aim to improve the classification performance of TTFS-based SNNs being accelerated on this work. The TTFS-based SNNs are constructed either by training from the scratch (Mostafa, 2016; Comsa et al., 2020) or by converting from the pre-trained ANNs (Rueckauer and Liu, 2018) have not yet been able to reach the classification performance of their ANN counterparts. As demonstrated in a recent study (Rueckauer and Liu, 2018), converting from ANNs to TTFS-based SNNs, unfortunately, leads to accumulated approximation errors, and results in a drop in accuracy. Our work tackles this problem by proposing a novel training approach to refine the network weights after conversion, which improves the accuracies of the converted TTFS-based SNNs. As we intend to train our SNN off-line and only perform inference on-chip, combining training on chip can lead to higher power consumption and lower energy efficiency. As a result, other neuromorphic accelerators with on-chip training capabilities (e.g., Loihi) are outside the scope of our work. The reason is that the hardware components used to support on-chip training in accelerators like Loihi result in additional area and power overheads during inference.

Overall, the main objective of this work is to accelerate SNN inference using TTFS-based solutions on low-power devices, with minimal loss of accuracy. Therefore, this work focuses on (1) improving the classification performance of TTFS-based SNNs, and (2) designing a low-power neuromorphic hardware accelerator for performing inference of TTFS-based SNNs. The main contributions of this work can be listed as follows:

- A new training algorithm that reduces the errors accumulated as a result of converting pre-trained neural network models to SNNs.
- An efficient mapping algorithm is proposed to achieve running different types of neural networks with high PE utilization.
- A novel low-power neuromorphic architecture, this work is designed to accelerate the inference operations of TTFS-based SNNs.
- An end-to-end neuromorphic technique that demonstrates the state-of-the-art performance and accuracy for TTFS-based SNNs.

The rest of this paper is structured as follows. Section 2.1 introduces SNNs and the existing algorithms for training TTFS-based SNNs. Section 2.2 introduces SNN hardware accelerators in detail. Section 2.3 describes the new training methodology model used to improve the accuracy of TTFS-based SNNs. Section 2.4 introduces the hardware architecture of the proposed TTFS-based SNN accelerator. Section 2.5 introduces the algorithms used to process spikes in this work. Section 2.6 describes the mapper used to accelerate the TTFS-based SNNs on this work. Section 3.1 introduces the experimental setup used to evaluate the proposed training algorithm and neuromorphic accelerator. Results and discussion on this work are then presented in Section 3.4. Finally, Section 4 concludes this paper.

## 2. Materials and methods

### 2.1. Background

In this section, we will introduce spiking neural networks (SNNs) in detail.

#### 2.1.1. Spiking neural networks

**Figure 1:**
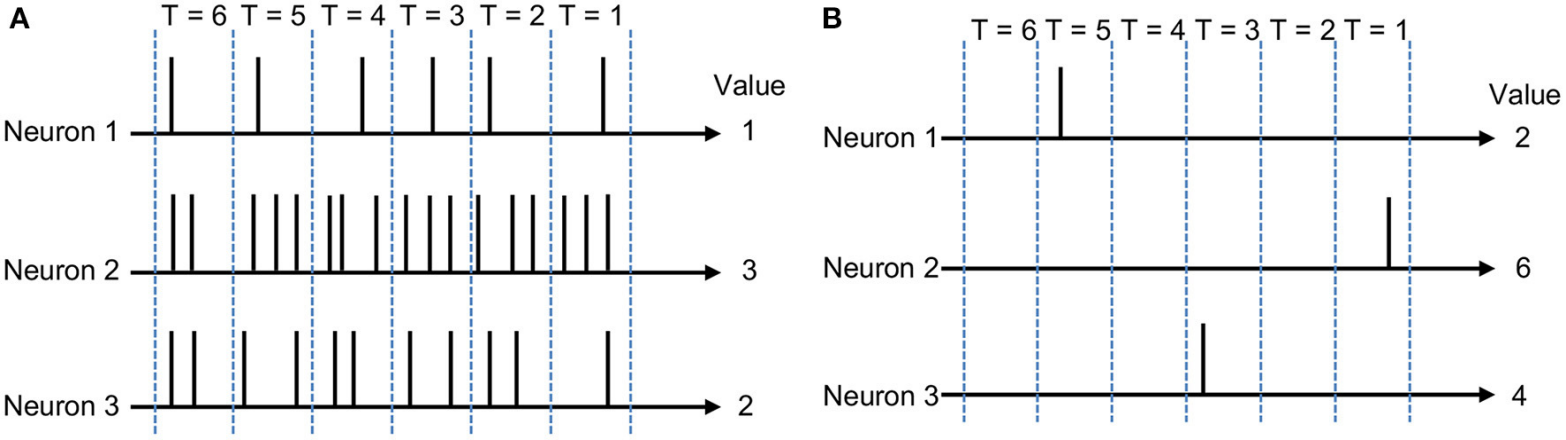
**(A)** Rate coding, where the mean firing rate of the neurons represents the information. For example, in **(A)**, the mean firing rate of Neuron 1 in each time step is 1. Similarly, the mean firing rate of Neuron 2 is 3 and the mean firing rate of Neuron 3 is 2. **(B)** TTFS coding, where the relative arrival time of the spikes with respect to the arrival time of the first spike represents the information. Generally, it takes longer to receive the spike if the information has less value. For example, in **(B)**, Neuron 2 receives the first spike in time step, T = 1. If the information represented by Neuron 2 is considered as 6, the information represented by Neuron 1 and Neuron 3 will be 2 and 4, respectively.

SNNs have garnered significant interest over the last few years, as a candidate for energy-efficient inference on low-power devices. In SNNs, the information is encoded in the form of discrete binary events called spikes, i.e., similar to the way the brain represents information. This is unlike ANNs where information is encoded as continuous values. The use of SNN processing reduces the computational power needed by using addition operations instead of the more power-intensive MAC operations used in ANNs. Furthermore, SNNs have very low neuron activation rates as compared to ANNs. Activations are low because every neuron can only be activated by a strictly positive input or a subset of all possible inputs above a pre-defined threshold. This translates to just a small subset of all neurons firing for any given inference. A small subset of neurons firing translates into a low memory access count, hence providing low power and energy consumption when performing inference. Furthermore, ANNs require synchronous tensor multiplication for each layer, while SNNs require asynchronous propagation of information. The two most prominent methods for propagating information through SNNs are rate coding (Gerstner et al., 2014) and temporal coding (Rueckauer and Liu, 2018). As shown in Figure 1A, in rate coding, the information is encoded by the mean firing rate of the neurons. Although there exist different definitions of firing rate, it often denotes either spike averaged over repetitions of an experiment or the average number of spikes over a period of time. This work refers to the latter when referring to rate-based networks. Rate-based networks become more accurate over time as more spikes are generated. From a power consumption point of view, each spike will require an associated memory access. Because rate coding has many spikes, having a weight look-up for each spike limits the minimum number of memory accesses and the corresponding amount of energy saved.

An alternative approach uses temporal coding to represent data in the neural network. The traditional temporal coding schemes include time-to-first-spike (TTFS; Mostafa, 2016), where information is represented by the relative time of arrival of the spikes with respect to the first spike (see Figure 1B), and phase-of-firing, where information is encoded using the time at which neurons fire within a periodic cycle (Cattani et al., 2015). When information is encoded in the TTFS scheme, neurons in an SNN only spike once during each inference pass and see many fewer spikes than their rate-based counterparts. By definition, the rate coding scheme can rely on the generation of multiple spikes over a fixed period of time for each value, while the TTFS coding scheme relies on the time taken for a single neuron to spike. Therefore, the TTFS coding scheme allows for fewer spikes than a rate coding scheme. Assuming that a spike corresponds to one memory access, the TTFS coding scheme allows for a low number of memory accesses. In addition, an inference pass of a TTFS-based network can end once the output layer produces its first output spike instead of waiting for the rest of the inputs to arrive. As a result, a minimal number of computations are performed for any particular inference, making temporal coding a highly suitable candidate for coding energy-efficient SNNs.

#### 2.1.2. Training of TTFS-based SNNs

Although significant power and energy savings can be achieved by using TTFS-based SNNs, TTFS-based SNNs that are constructed by either training from scratch (Mostafa, 2016; Comsa et al., 2020) or converting from the pre-trained ANN (Rueckauer and Liu, 2018; Lew et al., 2022) tend to not perform as well as their ANN counterparts in terms of the classification accuracy. As demonstrated in a recent study (Rueckauer and Liu, 2018), converting from ANNs to TTFS-based SNNs, unfortunately, leads to accumulated approximation errors, which results in significantly lower accuracy in the SNNs than the equivalent ANNs, particularly in larger network architectures. Our work aims to tackle this problem by proposing a novel training approach to refine the network weights after conversion, which improves the performance of the converted TTFS-based SNNs.

### 2.2. Related work

#### 2.2.1. Rate-based SNN accelerators

Though the above-mentioned neuromorphic hardware accelerators provide a promising pathway toward low-power and high-speed SNN acceleration, most of them were designed to emulate SNNs with rate coding schemes (Gerstner et al., 2014). In rate coding, the average number of spikes fired over a period of time represents the information. However, as processing each spike requires access to either on- or off-chip memory (i.e., to load the model parameters), the power consumed to accelerate the rate-based SNNs can be relatively high. For example, Shenjing (Wang et al., 2020) is a novel rate coding SNN accelerator. According to the results proposed by Guo et al. (2021), the number of spikes generated by rate coding can be over 4× more than the temporal coding. Therefore, under the same hardware structure design methodology, the rate-based SNN accelerators have to access the memory at least 4× more than the temporal coding SNN accelerator. Zhang's work (Zhang et al., 2021) and Skydiver (Chen et al., 2022) are recent rate-based SNN accelerators that all suffer from low energy efficiency due to a large number of spikes. Shenjing (Wang et al., 2020) also has to process a large number of spikes due to its rate coding scheme, which harms the energy efficiency. The mapping algorithm of Shenjing leads to a very large number of cores. Each output feature map has to be mapped on multiple processing elements (PEs). This idea of mapping will not only lead to a large number of cores but also generate a large amount of inter-core communication. Since one output feature map is divided into multiple parts which are mapped on separate PEs. Each PE has to send and receive partial sums generated by its neighbors. Shenjing's mapping algorithm introduces a large amount of data transfer between PEs.

#### 2.2.2. Temporal-based SNN accelerators

SpinalFlow (Narayanan et al., 2020) is a state-of-the-art temporal-based SNN accelerator. It processes a compressed, time-stamped, sorted sequence of input spikes. It adopts an ordering of computations such that the outputs of a network layer are also compressed, time-stamped, and sorted. All relevant computations for a neuron are performed in consecutive steps to eliminate neuron potential storage overheads. While SpinalFlow can achieve better energy efficiency with better data reuse, it suffers from three major drawbacks:

- First, the dataflow used by SpinalFlow is output stationary which is not the optimal traditional dataflow under high sparsity. Considering one convolutional layer with C input channels and N output channels. The size of each input feature map and output feature map is *W* × *H*. The size of the filter is *K* × *K*. Depending on the pattern of output stationary and input stationary, the number of accessing memory can be calculated mathematically. For the output stationary which is used by SpinalFlow, each neuron in the output feature map will access *K* × *K* × *C* input values and *K* × *K* × *C* weight values. Meanwhile, one neuron should be read and written. So the number of access of output stationary can be represented by Equation (1). Also, the number of access of input stationary dataflow can be represented by Equation (2).
  (1) $$\begin{array}{l} N_{output}=H\times W\times N\times \left(S_{p}\times K\times K\times C+K\times K\times C+2\right) \end{array}$$
  (2) $$\begin{array}{l} N_{input}=S_{p}\times H\times W\times C\times \left(K\times K\times N+1+2\times K\times K\times N\right) \end{array}$$
  Considering activation sparsity *S*_*p*_, the simplified of *N*_*input*_ < *N*_*output*_ is like Equation (3). This equation shows that when the sparsity is more than 0.5 the number of memory access generated by input stationary can be less than output stationary. Since the sparsity of TTFS-based SNN is relatively high (usually larger than 90%), the input stationary dataflow can be better for TTFS-based SNN.
  (3) $$\begin{array}{l} S_{p}<\frac{1+\frac{2}{K\times K\times C}}{2+\frac{1}{K\times K\times N}} \end{array}$$
- Second, the mapping algorithm has low PEs utilization which leads to low energy efficiency. SpinalFlow maps one *spine* in the output feature maps to 128 PEs. Each PE is responsible for producing a neuron in an output feature map spine. After computation, the neuron potential is compared to its threshold. If one neuron/PE has exceeded its threshold, it produces a spike. However, SpinalFlow takes advantage of TTFS coding which means one PE will be idle for the rest of the input interval because a neuron can only produce one spike in its input interval. This mapping algorithm will lead to low PE utilization. Further, PE utilization will become gradually lower because at each end of the time step every neuron will be compared with their threshold and then fire a spike depending on their neuron potential. With the time step increasing, more and more neurons will fire their spike which leads more and more PEs to be idle.
- Finally, beyond the limitations of the dataflow and mapping algorithm, the hardware design of SpinalFlow has missed a number of opportunities to improve its energy efficiency. One limitation of SpinalFlow is that it does not support pooling operations. This means that modern CNNs with pooling layers (Simonyan and Zisserman, 2014; Krizhevsky et al., 2017) cannot be run on the SpinalFlow, which can be limiting as many general-purpose CNNs make use of pooling layers. The other drawback is that SpinalFlow requires additional time and logic to reorder the input feature map buffers. Before processing the next spine, they must read 16 input feature map spines from the global buffer into 16 input feature map spine buffers. These 16 pre-sorted 128-entry spines need to be merge-sorted to produce the sorted 2 K entries that represent the input receptive field. Therefore, to initiate every step, 16 cycles are required to populate the input feature map.

### 2.3. Training competitive TTFS-based SNNs

As discussed earlier in Section 2.1, TTFS-based SNNs can be constructed by converting pre-trained ANN models into TTFS-based SNNs. However, the converted TTFS-based SNNs suffer from quantization errors that accumulate across layers which, compared to ANNs, can significantly reduce classification accuracy. A second source of error arises from the intrinsic differences in neuronal dynamics between spiking neurons in SNNs and analog neurons in ANNs. With a spiking post-synaptic neuron, an input spike coming from the synaptic connection with a large weight could drive the neuron's internal membrane potential to cross the firing threshold before subsequent inhibitory input spikes arrive. This problem can be explained by the different operating mechanisms of the spiking neuron and analog neuron. A spiking neuron integrates the temporally distributed input information over time, while an analog neuron responds to the input stimuli instantaneously. Raising the threshold value of the post-synaptic neurons may alleviate this problem. However, it is not the best option in practice since it adversely increases the latency for decision-making.

To address these problems, we propose a novel training method to systematically convert the pre-trained ANNs to more accurate TTFS-based SNNs. First, we apply a data-driven weight normalization strategy such that the neuron activation is not dominated by a few input spikes with large weights while also ensuring timely decision-making. Finally, to mitigate conversion errors, we propose a layer-wise training methodology. As a whole, the proposed training framework effectively closes the accuracy gap between the pre-trained ANNs and the converted SNNs.

#### 2.3.1. Firing threshold determination

Determining the right combination of neuronal firing threshold, weight, and bias values is crucial to strike a balance between classification accuracy and latency. Apart from the learnable parameters (weights and biases) that can be directly taken from the pre-trained ANNs, the firing threshold requires extra effort to be determined. An inappropriate threshold value will cause the converted SNN to perform significantly poorer than the equivalent ANN. One common approach to this problem would be to set the threshold to 1 and adjust the weights such that the activations are normalized. There are some works try to fix this by using dynamic thresholds or neuron potentials (Stöckl and Maass, 2021). But those solutions can require significantly more memory as each neuron now needs to have it's own threshold.

#### 2.3.2. Weight normalization

In order to prevent the converted SNNs from underestimating the output activation of the corresponding ANNs, this work applies weight normalization. One way to normalize the weights is to consider all possible combinations of positive activations that could occur at a particular ANN layer and scale the weights by that maximum quantity. The benefit of such an approach is that it only depends on the weights and biases of the network. However, in reality, the maximum activation that is determined in this way might be far from the actual activation values for the majority of neurons. This leads to weights and biases that are much smaller than what they need to be, increasing the time taken for a neuron to get activated. Because the time is taken for a neuron to first spike increases. A longer duration will be required to achieve high classification accuracy. This problem will be exacerbated in deeper networks if weights are normalized in this way for all layers.

Instead of this conservative approach, we estimate the maximum activation values of an ANN by making use of the training Images from the training set are converted into input spike trains before being propagated through a *L*-layered ANN to produce *n* sets of *L* activation vectors {**a^1^**...**a^L^**}, where each set contains the activation vectors obtained from propagating each of the *n* input spike trains. The scale factor for each layer, *s*_*current*_, is set to the magnitude of the maximum activation observed in {**a^1^**...**a^L^**}. Weight and bias vectors are then scaled by the scale factor. Note that because this algorithm uses data from the training set, a strong performance guarantee cannot be extended to the test set. As long as the training and test sets have a similar data distribution, which is typically the case, the activation vectors observed using the training set would be similar to that observed in the test set. However, there might be instances where the activations observed in the test set are more extreme than those observed in the training set, potentially leading to a reduction in accuracy. We feel that our proposal provides a better trade-off between latency and accuracy, as shown in Section 3.4.2.2. This is because the time taken to spike is shorter to achieve similar accuracy.

#### 2.3.3. Training network

Errors arising from converting ANNs to SNNs can be further reduced through (1) retraining an ANN with constraints or (2) refining the learnable parameters on the converted temporally-encoded SNN.

Training algorithms typically applied to SNNs can be broadly classified into two categories: (a) membrane potential driven and (b) spike driven. Membrane potential driven training algorithms treat membrane potentials as differentiable signals and use surrogate derivatives (Neftci et al., 2019), or use back-propagation through time (BPTT) (Wu et al., 2019) for training. While competitive results have been shown (Wu et al., 2019), these algorithms can be demanding in terms of memory and computation as the entire forward pass of the network needs to be stored to compute the necessary derivatives.

The second category is spike-driven learning algorithms which rely on the spike timing information to train the network. These algorithms (Shrestha and Song, 2017) usually assume that membrane potentials are linear around the time at which the neuron spikes to avoid dealing with non-differentiability. There are some works (Mostafa, 2016) that do not require this assumption by using integrate-and-fire neurons, which is one of the most widely used models for analyzing the behavior of neural systems.

Instead of choosing between training SNNs from scratch and converting ANNs-to-SNNs, we propose to convert an ANN to an SNN and train to minimize approximation errors. This allows for a significant reduction in the training time needed to construct an accurate SNN. We propose coupling each layer in an ANN and the corresponding layer in the converted SNN, and minimizing a layer-wise cost function. Unlike traditional SNN training algorithms which utilize a loss computed at the final layer, the algorithm we are proposing is aimed at minimizing the divergence between ANN activations *a*_*Li*_ and SNN activations *s*_*Li*_ for every neuron with index *i* in a layer *L*.

From the ANN-SNN conversion, the analog activation of a neuron in the ANN is equivalent to the instantaneous firing rate of TTFS-based SNN. The instantaneous firing rate is given by the inverse of the time taken for a neuron to first spike. It is possible to model the approximation between the activation of a single neuron *i* in a particular layer *l* in an ANN and the corresponding neuron in an SNN: $a_{i}^{l}=\frac{1}{t_{i}^{l}}+\epsilon$ where the introduction of ϵ allows for activation between SNN and ANN to deviate by a reasonable margin of error. A potential loss function is the L2-norm, given by $L=\frac{1}{2}*{\left(a_{i}^{l}-r_{i}^{l}\right)}^{2}$ where *r*_*li*_ is the instantaneous firing rate of neuron *i* in layer *l* given by $r_{i}^{l}=\frac{1}{t_{i}^{l}}$.

The loss function is minimized by updating synaptic weights as described in Algorithm 1. β is the fraction of neurons to keep. By removing the least salient 1−β neuron weights in *get*_*parameters*_*from*_*ann*, it is possible to remove connections that could potentially lead to long-latency spikes. For each iteration in all iterations *K*, the forward pass of ANN produces a set of activation vectors, {**a^1^**...**a^L^**}, and SNN produces a set of first time spikes vectors, {**t^1^**...**t^L^**}. For each layer, *L*, the divergence between the ANN activation vector and SNN instantaneous rate vector is computed and minimized. This has the effect of delaying or advancing spike times in the network. In Section 3.4, we demonstrate how this improved training method works to increase inference accuracy.

**Algorithm 1:**
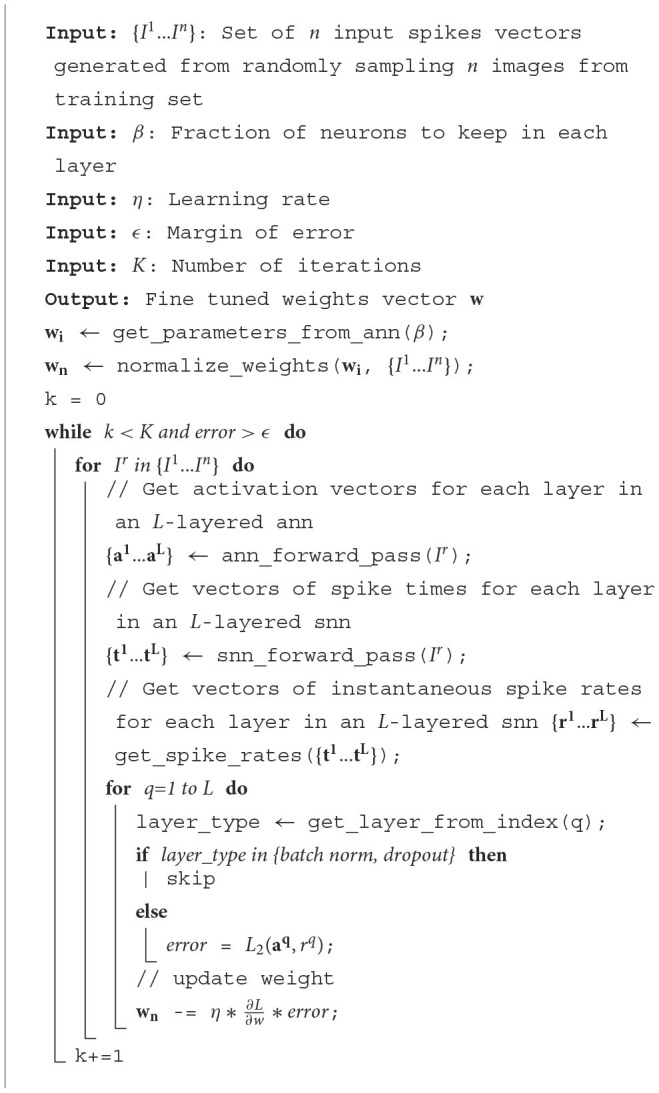
*train_network*: SNN training.

For complex datasets such as CIFAR or ImageNet, existing TTFS algorithms fail to produce high accuracy results. Although there are some temporal-based works that propose solutions with higher accuracy on CIFAR and ImageNet (Park et al., 2020; Stöckl and Maass, 2021), these spiking mechanisms require much more complex hardware design with much higher energy consumption. For example, high accuracy algorithms (Stöckl and Maass, 2021; Lew et al., 2022) require updating neuron potentials dynamically, which leads to complex logic and higher memory access counts. The original TTFS-based SNN algorithm, which is the main workload of this work, is currently unable to achieve comparable accuracy on these complex datasets. To achieve better accuracy, a new SNN mechanism, potentially using less-efficient methods, is required which is beyond the scope of this work.

### 2.4. Architecture description

**Figure 2:**
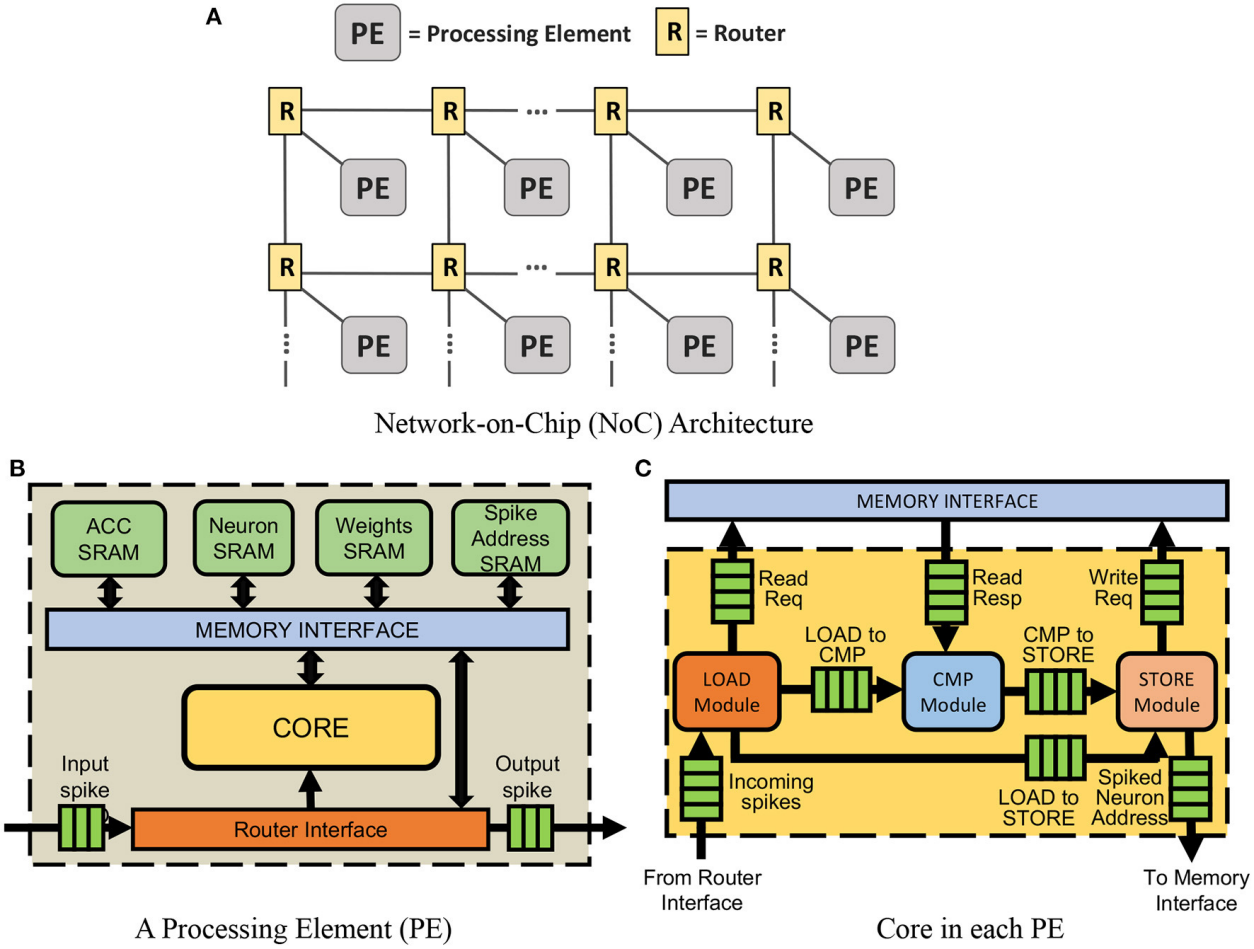
**(A)** The proposed accelerator with Network-on-Chip (NoC) architecture that is built on the OpenSMART NoC generator (Kwon and Krishna, 2017). The number of processing elements (PEs) required in the NoC depends on the size and complexity of the neural network being accelerated. **(B)** The architecture of each processing element (PE), and **(C)** core in each PE.

In this section, we describe the implementation details of our proposed hardware design and architecture. The objective of this work is to enable ultra-low-power inference operations using TTFS-based SNNs. As introduced in Section 2.1, during inference operations, TTFS-based SNNs can exhibit extremely low (sparse) spiking activity with small numbers of spikes being propagated and processed in the network (Guo et al., 2021). The aim of the accelerator design proposed in this work is to leverage the available sparsity in the network to optimize efficiency by minimizing the number of local memory accesses required per inference. We discuss the details of our implementation in the following subsections and Section 2.5, and an overview of the hardware architecture can be seen in Figure 2.

#### 2.4.1. Router interface

Input activations, or spikes, are received by the router interface, the gateway into the PE. It primarily performs three types of operations: (1) delivering the activation to the core for processing, (2) forwarding the activation to other PEs, and (3) receiving information from spike address SRAM when a neuron emits a spike and sends the spike to the destination processing element. In our design, operations (1) and (2) are performed at the same time to enable parallel processing of the activation.

#### 2.4.2. Memory interface

The memory interface consists of four individual SRAM interfaces—one for each of the SRAMs in the PE. This work organizes memory locally with computation units, which can reduce the latency of memory access and improve energy efficiency. This processing-in-memory nature helps this work outperform other traditional SNN accelerators. As this work uses single-port SRAMs to reduce power consumption, either a single reading request or a writing request can be processed at one time. As a result, the SRAM interfaces alternate between servicing requests from the Write Request FIFO and the Read Request FIFO to ensure that all the requests are processed within a reasonable amount of time.

#### 2.4.3. Core

The core is the key computational element of the PE. The design of our core is inspired by traditional deep learning accelerators (Luo et al., 2017; Moreau et al., 2018) and adopts a decoupled access-execute model (Smith, 1982). As shown in Figure 2C, It consists of three key modules: the load, compute, and store modules. The key idea adopted from the decoupled access-execute model is that all these three modules inside the core will only communicate with each other via FIFOs. As a result, the entire core is given some additional flexibility and will not be stalled immediately, even if one of the modules is stalled. It also enables the core to hide memory access latency due to the fact that the load module can compute target addresses and load target data values without waiting for the rest of the modules to be free. Meanwhile, the store module can push its store requests to the write request FIFOs, enabling it to generate additional store requests without waiting for its former store requests to be completed. This allows for better utilization of the core's resources as the other modules can continue execution if one of them encounters a stall.

##### 2.4.3.1. Load module

The load module (left unit in Figure 2C) processes three kinds of input: CNN input spikes, MLP input spikes, and End of Timestep (EoT) signals which are processed when all input spikes are received. There are three states in the load module:

*Configuration state*. At the beginning of receiving input spikes, the inner decoder will decode input spikes to initialize the registers used in the processing state. After configuring, the load module will come to the processing state.

*Processing state*. In this state, the load module will generate memory read requests with addresses that are sent to the corresponding on-chip SRAMs and the store module. Two of the generated addresses point to the corresponding weight and accumulated sum to be accessed from the weight SRAM and ACC SRAM in the computation of a spike in the compute module. When a CNN input spike is received, generated addresses to access the weight and accumulated sum are relevant to the coordinate of the spikes and the size of the filters. When an MLP spike is received, weight and accumulated sum access addresses are generated based on the neurons that are connected with the spike. A neuron address is generated when EoT is received and a read request is sent to the neuron SRAM to access the neuron potential. After processing a spike, the load module sends a read signal to the input FIFO to read a new input spike. Then the load module transits to the idle state until the next input is read from the FIFO.

*Idle state*. In this state, the load module will stay idle until receiving a new input spike read from the input FIFO again. Then the load module will enter the configuration stage again.

##### 2.4.3.2. Compute module

Spike data is accumulated in the compute module. Like the load module, the compute module (center unit in Figure 2C) is a finite state machine with an idle state and an active state. In the idle state, the compute module waits for the load module to send the spike type and the number of neurons that connect with the input spike. The compute module initializes its registers with this data and transits to the active state.

In the active state, the compute module performs addition operations on the data retrieved from the memory interface in response to the memory read requests generated by the load module. When a CNN or MLP spike is received, the compute module accumulates weights into the accumulated sum. When an EoT spike is received, the compute module accumulates accumulated sums to neuron potentials. Saturating adders are used to prevent overflow and underflow. The results are then sent to the store module along with the spike type. After all the data is processed, the compute module transits back to the idle state.

##### 2.4.3.3. Store module

The store module (right unit in Figure 2C) has two states: active state and idle state. In the active state, the store module can process three kinds of input: results from compute module, the EoT signal, and the softmax signal.

The store module can take the results generated from compute module and store them in the address obtained from the load module. When the core is processing the CNN or MLP input spikes, the store module takes the store address from the load module and the accumulated result from the compute module. In this stage, the store module only needs to store the value to the accumulated SRAM.

When the store module receives an EoT signal, it will check the value of neuron potential (the result computed and sent from the compute module) first. If the neuron potential is greater than the threshold set during the programming stage, the store module will send a spiking signal to spike SRAM. The corresponding neuron stored in spike SRAM whose potential is greater than the threshold will then be sent as a spike to the next layer. There is also a max-pooing mask in the store module. In layers where max-pooling is required, the store module first applies a max-pooling mask to the address of the incoming neuron potential. Then, it identifies neuron potentials that are in the same pool in the max-pooling process. From the pool, only the spiking signal of the max neuron potential is sent to the spike SRAM.

After receiving the softmax signal, the store module will compare neuron potentials in this layer. Then it will find the maximal value of neuron potential in this layer and the neuron address. This address will be sent to the spike SRAM.

### 2.5. Dataflow

#### 2.5.1. Convolutional layers

We will now discuss the algorithms used to process spikes in this work. Each PE in our hardware supports the processing of two types of spike signals—input spikes and End-of-Timestep (EoT) signals. Input spikes are the spikes generated by traditional integrate-and-fire neurons in SNNs, and EoT signals are the spikes used to indicate that a timestep is complete. In this work, to differentiate between these two spike signals, an additional bit is appended to all the spike packets as a prefix. If the prefix of the spike packet is “0,” it is treated as an input spike and processed accordingly. If the prefix of the spike packet is “1,” it is treated as the EoT signal.

When a PE in our hardware receives an input spike, it will be processed using the spike processing algorithm described in **Algorithm 3**. This algorithm uses eight registers to implement an access pattern for accessing the weights required and calculating the accumulated weights. Two registers store the addresses of weight (w_address) and accumulated weight (acc_addrs) to be accessed. Two register stores the values of variable, *x* and *y* in **Algorithm 3**. Two registers store the offsets of this input (i.e., referred to as y_jump and x_jump in **Algorithm 3**). The last two registers store the width of the output feature map and weight, wid_output, and wid_weight in **Algorithm 3**.

For example, let us consider one convolutional neural network (CNN) with one convolutional layer containing six by six neurons, three by three weights, and a two by two max-pooling layer. This simple CNN can be mapped on one PE. This PE is required to process the input spikes. The addresses 0–8 in weights SRAM will store the weights. The accumulated weights will be stored at the addresses 0–35 in the accumulated weights SRAM in the PE assigned for the convolutional layer. In **Algorithm 3**, the START_Acc and START_Weight will be different values depending on the position of the input spike in the input image. Assuming that the padding in this CNN is one, the START_Acc of input in position (0,0) will be 7, and the START_Weight should be 0.

If neuron *j* in the convolutional layer emits a spike, the acc_addrs and w_address in the second layer PE will be set based on the index of *j*. The core will then generate the required number of memory accesses depending on the values of x_jump, y_jump, START_Acc, and START_Weight. For instance, if the first neuron in the first convolutional layer emits a spike to the next layer in time step, *t*_*i*_, the spike packet emitted contains x_jump = 1, y_jump = 1, START_Acc = 7, and START_Weight = 0. When the core receives this packet, weights at the address 0 will be read from the weights SRAM first and added to the accumulated weights. According to the **Algorithm 3**, acc_addrs will then be updated to 6 and w_address will be 1. After that, x will be equal to x_jump. Thus, the inner loop will be complete. acc_addrs and w_address will be recalculated. In the second round of the inner loop, acc_addrs should start from 1 and w_address should start from 3.

At the end of *t*_*i*_, the accumulated weights will be added to the neuron potentials. If neuron potentials cross the threshold, output spikes are generated and transmitted to the next layer. Thus, using this new algorithm, our core can accelerate CNN efficiently without unnecessary computations and memory accesses.

Next, as time is used as the state variable to process information in this work, EoT signals are used to indicate that a timestep is complete and the accelerator can move on to the next time step. The PEs in our hardware support two ways of handling EoT signals—the standard integrate-and-fire method and the softmax method, which is normally used in the final layer of the networks.

**Algorithm 3** also shows how a PE updates neuron potentials when it receives an EoT signal. When the type of layer is the fully connected layer or convolutional layer without max-pooling, each neuron loads a neuron potential and its accumulated weight, adds the neuron potential with the accumulated weight, and stores the result back to the neuron potential address. In the standard integrate-and-fire method, spikes are generated when the neuron potential crosses the threshold and if the neuron has not spiked before. But for the convolutional layer with a max-pooling layer, there is a max-pooling mask to help the PE knows which neurons should emit a spike after max-pooling. If one neuron's potential is more than the threshold and this neuron has not spiked before, the PE checks the max-pooling mask of this neuron. If this neuron is not marked in the mask, PE should mark this neuron and all neighbors of this neuron in the mask. The position and the number of neighbors depend on the size of the max-pooling layer. After marking the mask, this neuron can emit a spike.

#### 2.5.2. Fully connected layers

##### 2.5.2.1. Processing of input spikes

When a PE in the accelerator receives an input spike, it will be processed using the spike processing algorithm described in **Algorithm 3**. This algorithm uses four registers to implement an access pattern for accessing the weights required and calculate the accumulated weights, which are the gradients of neuron potentials as introduced in Section 2.4. The first register stores the addresses of weight (w_address) and accumulated weight (aw_address) to be accessed. The second register stores the value of variable, *k* in **Algorithm 3**. The third register stores the number of neurons assigned for that PE (i.e., referred to as x_jump in **Algorithm 3**). The fourth register stores the amount of increment in aw_address and w_address after each weight access (i.e., referred to as x_inc in **Algorithm 3**).

If neuron *j* in the first layer emits a spike, the aw_address and w_address in the second layer PE will be set based on the index of *j*. The core will then generate the required number of memory accesses depending on the values of x_jump and x_inc. For instance, if the second neuron in the first hidden layer emits a spike to the next layer in the time step, *t*_*i*_, the spike packet emitted contains a weight offset, W_START = 4. When the core receives this offset, weights at addresses 4–7 will be read from the weights SRAM and added to the accumulated weights. As we will discuss in Section 2.5.2.2, at the end of *t*_*i*_, the accumulated weights will be added to the neuron potentials. If neuron potentials cross the threshold, output spikes are generated and transmitted to the next layer. Thus, using this algorithm, our core can accelerate FCNs efficiently without unnecessary computations and memory accesses.

##### 2.5.2.2. Processing of end-of-timestep (EoT) signals for softmax layers

For MLP layers, the algorithm for EoT handling for the integrate-and-fire layer is the same as the Convolutional layer without max-pooling in Algorithm 2. For the softmax layer, only the neuron with the largest neuron potential produces a spike. In both methods, an EoT signal is sent to the next layer after the last PE in the current layer has finished updating the neuron potentials and generating spikes.

**Algorithm 2:**
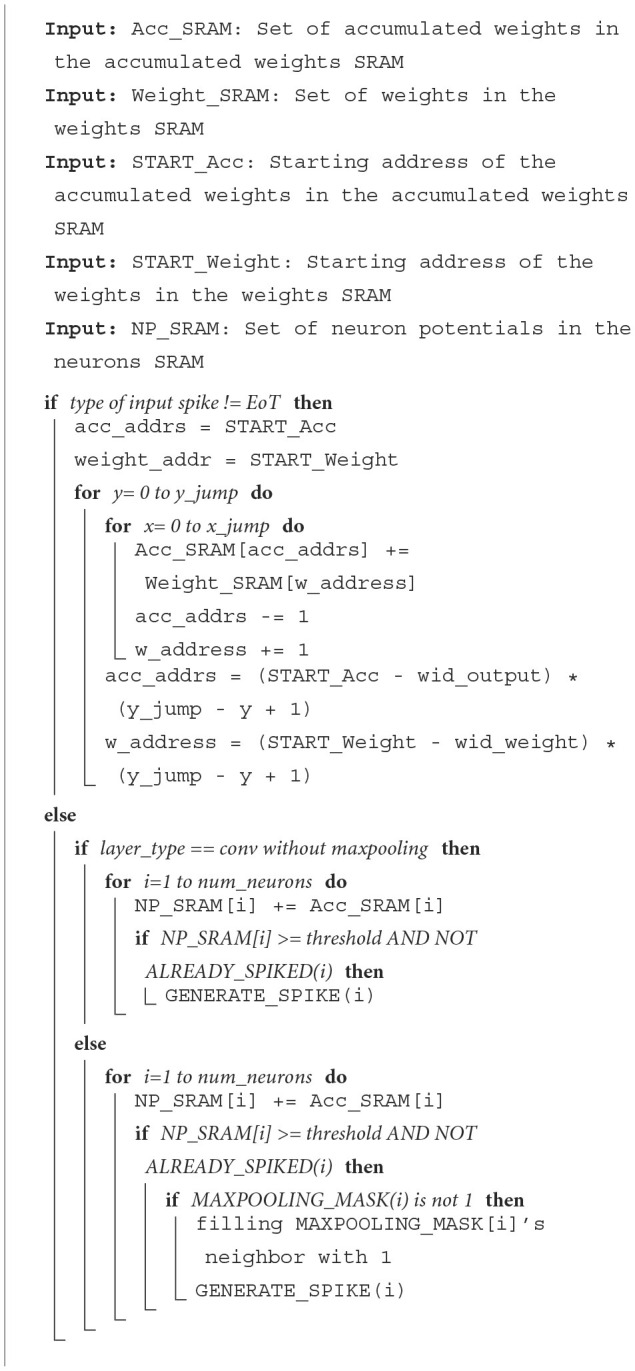
Input spike processing algorithm.

**Algorithm 3:**
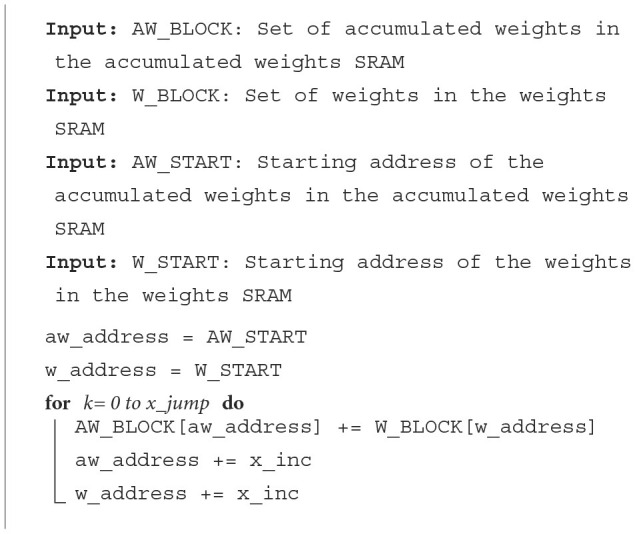
Spike processing algorithm.

**Algorithm 4:**
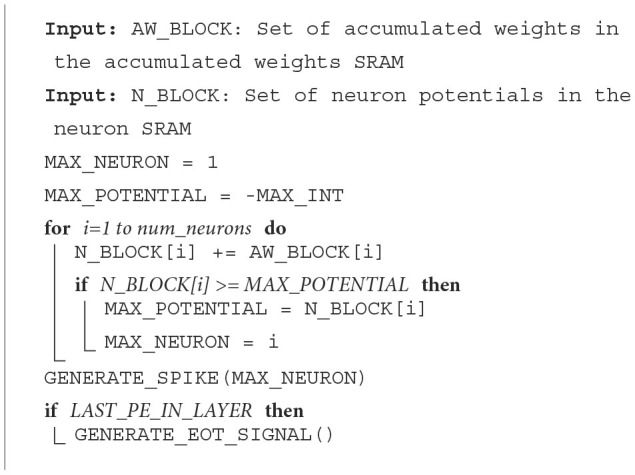
Algorithm for EoT handling for softmax layers.

### 2.6. Mapping methodology

#### 2.6.1. Convolutional layers

Before running, the mapper needs a trained CNN model from the training software The mapping technique proposed helps eliminate the need for memory transactions between different PEs.

In this section, we explain how SNNs are mapped to our accelerator using the mapper that enables efficient computations on our accelerator. Algorithms for mapping convolutional, max-pooling, and fully connected layers are explained here.

To map an *N* × *N* convolutional layer, a minimum of $CC=MAX\left(\frac{n}{N},\frac{w\times w\times c}{W}\right)$ PEs are needed where N and W are the maximum numbers of neurons and weights that can be mapped to a single PE, respectively, where *w* represents the width of the filters and *c* is the number of channels mapped to different PEs. As the max-pooling layer is always used in the convolutional layers, each PE that is mapped to compute a convolutional layer contains a configurable max-pooling layer.

As CNNs can also require fully connected layers as the last stages of a network, a mapping algorithm that maps MLPs to our accelerator is needed. To map a *m* × *n* fully-connected layer, a minimum of $CF=MAX\left(\frac{n}{N},\frac{m\times n}{W}\right)$ PEs are needed, where *N* and *W* are the maximum numbers of neurons and weights that can be mapped to a PE. The PEs are placed within a $\sqrt{CC+CF}$ by $\sqrt{CC+CF}$ grid. One PE can only be allocated to one layer to process all spikes received by this layer.

#### 2.6.2. Parameter mapping

The parameters to be mapped to our accelerator consist of biases, weights, and output addresses.

##### 2.6.2.1. Neurons and bias

For a convolutional layer, the mapper allocates one of the channels in at least one PE. To avoid memory transactions between different PEs, the number of neurons mapped on one PE should be multiple of the size of the max-pooling layer. In this way, the neurons needed to be processed by the max-pooling layer are in the same PE. For example, to map a 28 × 28 × 2 convolutional layer with one 2-max-pooling layer, the maximum number of neurons on one PE is 256. Since the maximum number of neurons that can be mapped to one PE is 256, each 28 × 28 feature map has to be mapped to at least 4 PEs. Meanwhile, each channel has to be mapped to at least one PE. Therefore, this 28 × 28 × 2 layer will be mapped on at least 4 × 2 PEs. For a fully connected layer, the number of neurons mapped depends on the maximal number of neurons and weights in the PE. Take one fully connected layer with 128 neurons and 1,568 inputs as an example. Assuming that the PE can store at most 9K weights and 256 neurons, these 128 neurons will be mapped to $22=MAX\left(\frac{128}{256},\frac{1568\times 128}{9,000}\right)$ PEs.

In the convolutional layer, the values of the biases are different between channels. Mapper takes biases from CNN trained model and then converts these biases to the format of hardware compatible. For the fully connected layer, each neuron has its own bias value. The mapper maps biases in the trained model to each neuron in the fully connected layer.

##### 2.6.2.2. Weights

**Figure 3:**
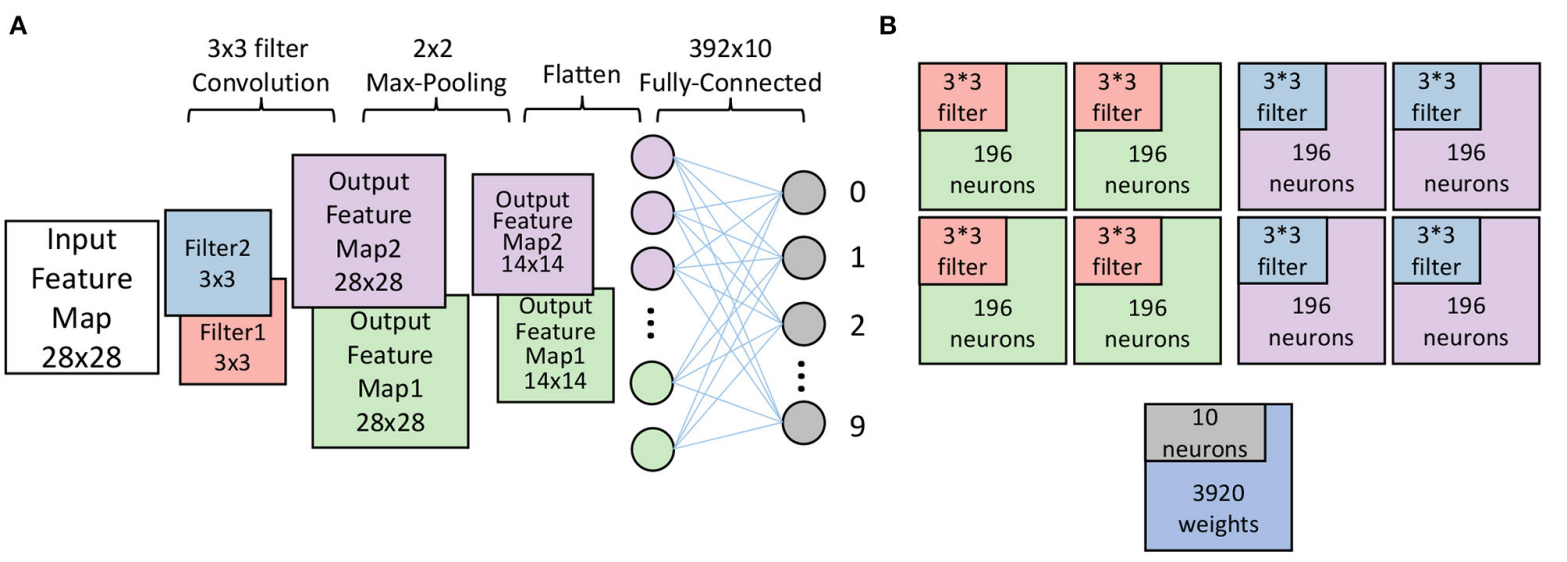
**(A)** A toy CNN with one convolutional layer, one max-pooling layer, and one fully-connected layer. **(B)** The cores used to map the CNN in **(A)**. Two channels in the convolutional layer are mapped to eight PEs with relevant filters. The fully-connected layer is mapped on one PE. The different colors represent the different parts of the CNN.

Weights in the convolutional layer (also called a filter) have to be mapped channel by channel. As mentioned before, one PE can only be allocated to one layer. It means that one layer can be mapped to multiple PEs, which improves the scalability of our design. It also applies to channels. When one channel is mapped to multiple PEs, each PEs mapped to this channel will be allocated the same filter. As for the fully connected layer, one input spike of one fully connected layer will update all neurons in the fully connected layer. Hence, one PE which contains *m* neurons will store *m* × *n* weights in its weights SRAM, and *n* is the number of input spikes of this fully connected layer. According to this access pattern in hardware, weights associated with corresponding neurons will be mapped on one PEs. As shown in Figure 3B, the mapper we proposed mapped a CNN whose structure is shown in Figure 3A. The mapper maps each feature map to four cores according to the rules proposed in Section 2.6.2.1. Since each feature map shares the same filter, each core stores the corresponding filter separately (represented by a different color).

##### 2.6.2.3. Input

Input data of this work can be various kinds of data such as imagines from MNIST or Fashion-MNIST, features generated by front-end processors, audio signals, etc. The mapper converts these input data items into a form that can be used by our custom hardware design. There are two kinds of CNN data format packets that are used in our accelerator. The mapper divides the input into several timesteps according to the value of each input value. The input in the same timestep represents the same value. The information stored in each input spike is about weights and the neuron this input will access. For CNN format spikes, one converted input spike contains information about the weights address and neurons address where this spike starts accessing and the number of weights and neurons the spike will access. The CNN format input spike is organized by this format:


{Ch,Y jump,X jump,Sneuron,Sweight}

**Figure 4:**
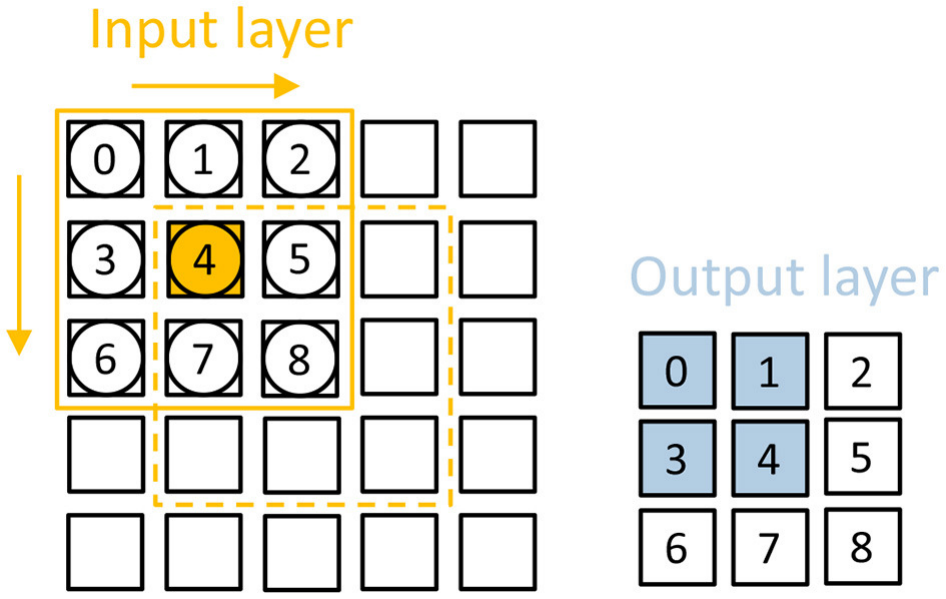
A simple example that explains the dataflow. There is one input feature map on the **left** and one output feature map on the **right**. When one input activation is fired (which is the box in orange.), weights 0/1/3/4 are read to update neuron 3/4/0/1 in the output feature map.

*Ch* is short for Channel, which means the channel this pixel belongs to. The *Yjump* and *Xjump* represent the way the filter should move on this pixel, which means that the filter should move *Xjump* times in the direction of the x-axis and move *Yjump* times in the direction of the y-axis. The *Sneuron* means the start neuron this input spike should access at first. It's the same with the *Sweight*, which is the first weight this input should access. Once the load module has this information, it can load values from addresses computed by these parameters. As each input spike will update relative accumulated addresses, the input spikes also contain the information on padding and stride. As shown in Figure 4, the size of the input is 5 × 5 and the size of one output feature is also 5 × 5. A 3 × 3 filter is used with the 1 stride and without padding. For the first pixel in the input whose index is (0,0), the input spike should be:


{Ch(0),Y jump(1),X jump(1),Sneuron(4),Sweight(0)}

This input spike means that it will access neuron 4 (1,1) and weight 0 (0,0) as the first neuron and weight address.

If a spike is encoded in MLP format, this spike will store the weight address which is the start address accessed by this spike. As mentioned in Section 2.6.2.2, one input will update all neurons in the fully-connected (FC) layer with corresponding weights. Hence the information stored in the spike will give the start address of the corresponding weights.

##### 2.6.2.4. Spiking address

Spiking addresses mean that when one neuron's potential is more than the threshold, the value is fired to the next layer. Thus the value of spiking addresses can be seen as the input of the next layer. In the convolutional layer, the value of spiking addresses depends on the position of each neuron in the feature map. They can be seen as the pixel in the input image. As the spike addresses are the input of the next layer, the spiking address has the same format as the input spike. The mapper will generate the value of the spiking address depending on the type of the next layer and how neurons connect with the next layer.

##### 2.6.2.5. Programming spikes

This kind of spike contains various information needed for each PE. For each PE, the width of the filter and output feature map, the value of threshold and max_time_steps, and the type of the layer mapped on the PE will be set before running the whole accelerator. In addition to these parameters, the first PE in each layer is chosen to be the output destination PEs. The forwarding destinations also need to be set on each PE.

#### 2.6.3. Fully connected layers

In this section, we introduce the mapper used to accelerate the TTFS-based SNNs on this work.

To accelerate FC layers on our accelerator, the mapper enables us to calculate the minimum number of PEs needed for accelerating a particular neural network, depending on the number of layers in the network, neurons, and weights in each layer. For example, to map *m* × *n* fully-connected layers, a minimum of $C=MAX\left(\frac{n}{N},\frac{m\times n}{W}\right)$ PEs are needed, where *N* is the maximum number of neurons that can be mapped to a single PE, and *W* is the maximum number of weights that each PE can store. As shown in Figure 2, the PEs are placed within a $\sqrt{C}$ by $\sqrt{C}$ grid.

The mapper also assigns a layer (or a group of neurons in a layer) to each PE in the grid. To optimize for latency, more PEs can be assigned to a particular layer. It also facilitates the transfer of learned model parameters from software to hardware by converting the biases, weights, and initial neuron potentials of the network into spikes as required by this work. The mapper also generates the spikes needed to fill the values of xjump and xinc in the respective registers, as we discussed in Section 2.5.

## 3. Results

### 3.1. Experimental methodology

In this section, we outline the details of the experimental setup and algorithms used in evaluating our work.

### 3.2. Networks

The MNIST Handwritten Digits dataset (LeCun et al., 1998) contains gray scale images of 10 handwritten digits of size 28 × 28, with a total training set of 60,000 examples, and a test set of 10,000 examples. We built an FCN with four layers (784-300-300-10), i.e., with the input layer containing 784 neurons, two hidden layers containing 300 neurons each, and an output layer containing 10 neurons. We also built some CNN workloads, which are shown in Table 1. These networks are trained offline using the training methodology introduced in Section 2.3. The learned weights and biases are then transferred to our hardware for accelerating the inference operations. Usually, quantized weights and biases are used to achieve high energy and area efficiency in neuromorphic accelerators. In this work, we quantized the weights and biases to 8-bit precision before transferring them to our hardware. Timestep is set to 8 in TTFS coding SNN. We also studied the influence of such quantization on the classification accuracy of our network.

**Table 1:** Workload used for comparing with different accelerators.

**Shenjing-CNN**	**Systolic-CNN-a**	**Systolic-CNN-b**	**SC**	**DWC**	**PWC**
Input (28, 28, 1) Conv1 (3, 3, 1, 16) Pool1 (2, 2) Conv2 (3, 3, 16, 32) Pool2 (2, 2) FC1(1568, 128) FC2 (128, 10)	Input (28, 28, 1) Conv1 (5, 5, 1, 12) Pool1 (2, 2) Conv2 (5, 5, 12, 64) Pool2 (2, 2) FC1 (3136, 10)	Input (28, 28, 1) Conv1 (5, 5, 1, 64) Pool1 (2, 2) Conv2 (5, 5, 64, 64) Pool2 (2, 2) Conv3 (5, 5, 64, 100) FC1 (4900,10)	Input (14, 14, 64) SC (3, 3, 64, 64)	Input (14, 14, 1) DWC (3, 3, 1, 64)	Input (14, 14, 512) PWC (1, 1, 512, 512)

SC, standard conv; DWC, depth-wise separable conv; PWC, point-wise separable conv.

### 3.3. Hardware simulation environment

Our accelerator is synthesized using Synopsys Design Compiler version P-2019.03 targeting an industry-grade 22 nm technology node. Gate-level simulations are performed using Synopsys VCS-MX K-2015.09 and power analysis was performed with Synopsys PrimePower version P-2019.03. The simulations are performed at different frequencies to achieve comparable latency with other designs and account only for inference time. Table 2 shows the system configuration used.

**Table 2:** Hardware configuration parameters.

**Name**	**Size**
Tech node	22 nm
Accumulated weights	1 kB
Neurons	1 kB
Weights 1	9 kB
Weights 2	19 kB
Spike address	2 kB

Weights 1 is for the MNIST MLP model and CNN models. Weights 2 is for the Fashion MNIST MLP model.

### 3.4. Results and discussion

In this section, we first describe the accuracy, power, and energy efficiency results, and finally analyze the impact of noise.

#### 3.4.1. Comparison with SpinalFlow

To present a fair comparison with SpinalFlow, we aim to run the same workload proposed in their paper (SC, DWC, and PWC in Table 1) on our design and compare our results with their proposed results. Activation sparsity levels are also set to be the same as evaluated by SpinalFlow, which is the ratio between zero value neurons and the total number of neurons. For example, Sp60 means that 60% of the neuron potentials are zero. We compare our work with SpinalFlow separately because SpinalFlow is modern temporal coding SNN accelerator. It is worth clarifying how our work aims to improve over this (and other) previous works. Furthermore, SpinalFlow focuses mainly on optimizing its hardware design rather than the SNN algorithm. Hence, we begin by comparing the same workload used in the show the benefits of our hardware implementation. We then compare our work with other SNN accelerators on both hardware performance and accuracy in Section 3.4.2.

##### 3.4.1.1. Latency

**Figure 5:**
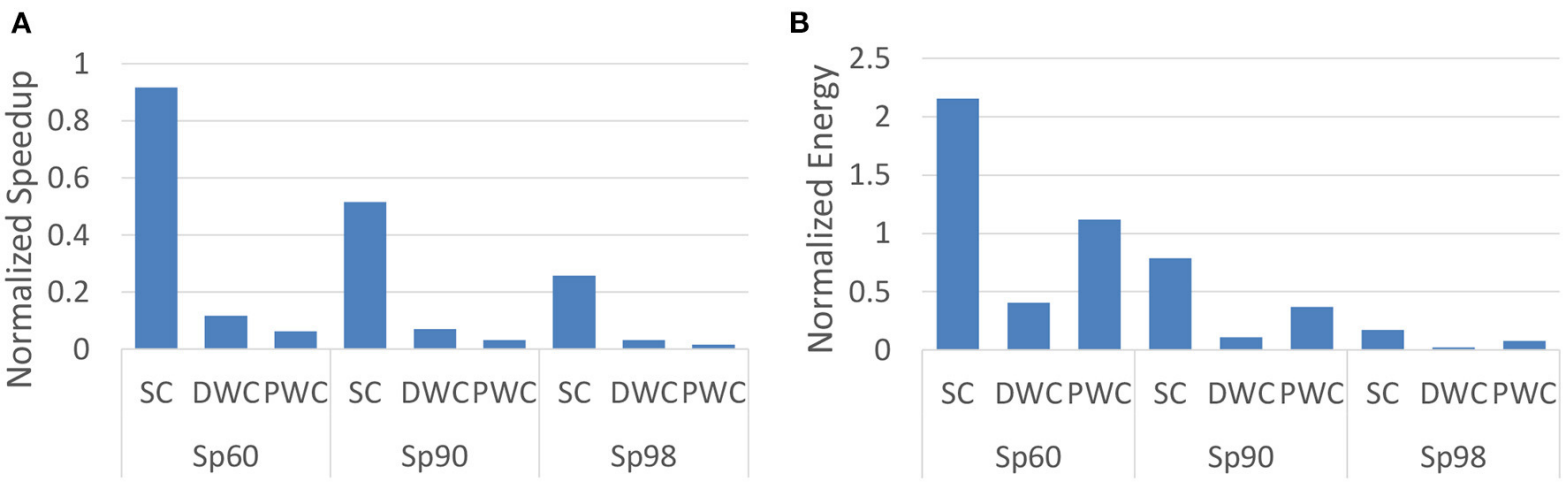
Comparison with SpinalFlow on latency/inference and energy/inference by running workload SC, DWC, and PWC. Sp is activation sparsity, which means the ratio of non-zero input activations to the total number of input activations. Sp60, Sp90, and Sp98 refer to 60, 90, and 98% activation sparsity for the SNN. **(A)** Speedup normalized to SpinalFlow. **(B)** Energy normalized to SpinalFlow.

The latency per inference, with a batch size of 1, for this work is normalized to SpinalFlow as shown in Figure 5A. At high activation sparsity levels, in this example is set to 98%, this work can operate 3.88×, 31.35×, and 64.6× faster than SpinalFlow. Even at relatively lower sparsity (60%), this work is still 1.09×, 8.5×, and 16.2× faster on SC, DWC, and PWC workloads. Different from SpinalFlow, which maps each neuron of one spine to each PE, this work maps the entire output feature map to one PE to enable efficiency with high utilization. Whenever one input spike comes to the PEs, all PEs will be active. This mapping algorithm leads to a near-100% PE utilization.

##### 3.4.1.2. Energy consumption

To evaluate the energy efficiency of the this work and to compare against SpinalFlow, we evaluate the workloads proposed by SpinalFlow at different levels of sparsity. These workloads are shown in Table 1. Based on our mapping algorithm and the size of the output feature map, each channel is mapped to one PE. Figure 5B compares the energy consumption of this work and SpinalFlow on different workloads. It can be seen that this work achieves 5.7×, 45.9×, and 12.9× lower energy consumption at very high sparsity levels (98%).

#### 3.4.2. Performance and power consumption

##### 3.4.2.1. Accuracy

**Figure 6:**
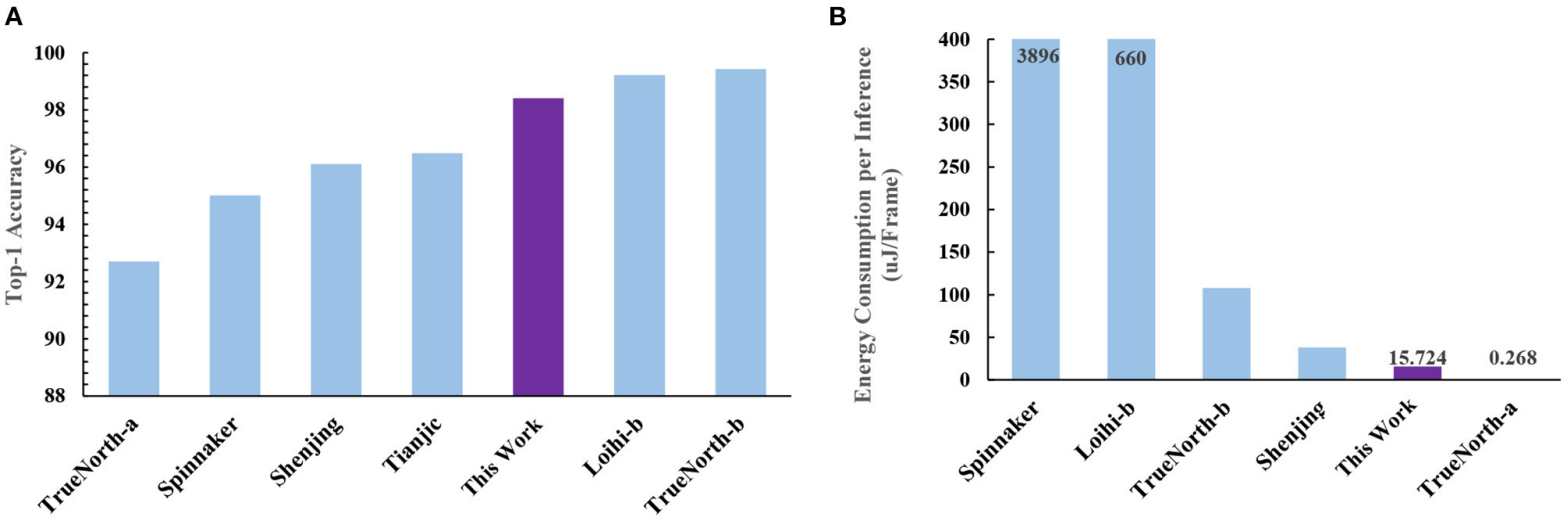
A comparison to other neuromorphic accelerators based on **(A)** accuracy and **(B)** energy consumption per inference. This work is the only one that achieves both high accuracy and low-energy consumption at the same time. TrueNorth-b consumes almost 6.87× more energy than this work to achieve its higher accuracy, while TrueNorth-a gives up a significant amount of accuracy (92.70 vs. 98.40% for our work) to achieve lower energy consumption.

Prior TTFS-based work (Rueckauer and Liu, 2018) has shown an accuracy of 98.30% (without quantization) on the MNIST dataset. Our work, in contrast, achieves an accuracy of 98.44% (without quantization) and 98.40% (with quantization; see Table 3 and Figure 6A). Our proposed training method improves the accuracy of fully-connected TTFS-based SNNs on the MNIST dataset than previous TTFS-based SNN accelerators (by 0.85%; Mostafa, 2016) and most other rate-based SNN works. While the rate-based accelerator, TrueNorth-b, reaches a slightly higher accuracy than this proposed work, the costs are significant, with almost 3.83× lower energy efficiency and a 147× higher power consumption.

**Table 3:** Classification performance on MNIST dataset.

**Network**	**Coding**	**ANN acc (%)**	**SNN acc (%)**
TrueNorth-a	Rate	–	92.70
TrueNorth-b	Rate	–	99.42
Mostafa	Temporal	–	97.55
Comsa et al.	Temporal	–	97.96
Rueckauer et al.	Temporal	98.56	98.30
**This work**	Temporal	98.56	**98.44**
**This work + Quant**.	Temporal	98.56	**98.40**

The bold values indicated to highlight the results.

We also evaluate the performance of this work on relatively complex datasets, such as Fashion MNIST (Xiao et al., 2017). A simple three-layer MLP (784-1000-10) and a convolutional neural network (CNN) with two convolution, two max-pooling, and two fully connected layers are used for the study of the Fashion MNIST data (see Table 4). These networks are trained as ANNs, converted to SNNs, and retrained using Algorithm 1. In a similar way to previous works (Esser et al., 2016; Yakopcic et al., 2017), we convert CNNs to MLPs to accelerate them on our accelerator. The comparison between the accuracy of this work and other works on Fashion MNIST data is shown in Table 4. This work provides good classification performance with minimal accuracy drop from its ANN equivalent and high energy efficiency compared to the state-of-the-art works. Meanwhile, the number of spikes in our work is 4.8× lower than the rate-based coding SNN. However, we observed an accuracy drop (5.1%) between this work and its equivalent ANN. The reason is that this work has more layers and when the number of layers in the network increases, the ANN-to-TTFS-based SNN conversion error increases.

**Table 4:** Classification performance on fashion MNIST dataset.

**Proposal** **(Network architecture)**	**Coding**	**No. of spikes**	**ANN acc (%)**	**SNN acc (%)**
S4NN (784-1000-10)	Temporal	–	–	88.00
**This work (MLP)** **(784-1000-10)**	Temporal	128	88.78	**88.21**
**This work** **(28×28-16C3-P2-32C3-P2-128-10)**	Temporal	–	91.71	**86.50**
Zhang et al. (784-400-400-10)	Rate	621	–	89.50
Hao et al. (784-6000-10)	Rate	–	–	85.30

The bold values indicated to highlight the results.

##### 3.4.2.2. MNIST

With the TTFS-based accelerator proposed in this work, the power and energy consumed per inference are significantly lower than that of the vast majority of rate-based accelerators. Table 5 shows the storage space needed and weight data width used by other work. Table 6 and Figure 6B show the performance on the MNIST dataset. To achieve comparable latency, the simulations are performed at 18 MHz and 720 KHz when comparing with Systolic SNN-CNN-a and Shenjing-CNN, respectively. For comparison with other works, the simulation is run at 120 KHz. It should be noted that the latency only accounts for the inference time.

**Table 5:** Storage and weight width configuration of other SNN accelerators.

**Accelerator**	**Storage/Core**	**Weight width**
Systolic SNN	–	32b
Shenjing	–	5b
Spinnaker	96.00 KB	32b
Loihi	264.00 KB	9b
TrueNorth	12.75 KB	4b
Tianjic	125.00 KB	8b
SpinalFlow	4.50 KB	8b
This work	13.00 KB	8b

**Table 6:** Comparison of this proposal with rate-based neuromorphic accelerators on the MNIST dataset.

**Accelerator**	**Enc**.	**Acc**.	**FPS**	**Tech**	**Power**	**uJ/Frame**	**No. of cores**	**Power/Neuron**	**Network**
Systolic SNN-CNN-a	Rate	98.98	333	–	754.00	2237.24	1024	-	CNN
This work	Temp.	98.90	333	22	53.49	160.63	113	1.85	
Shenjing-CNN	Rate	97.15	30	28	87.54	2918.00	705	0.49	CNN
This work	Temp.	97.90	30	22	3.20	106.51	120	0.10	
Spinnaker	Rate	95.01	77	130	300.00	3896.00	48	1.20	MLP
Shenjing-MLP	Rate	96.11	40	28	1.26	38.00	10	0.49	
Systolic SNN-MLP	Rate	98.84	278	–	745.00	2679.86	1024	–	
Zhang et al.	Rate	95.30	3,704	–	–	340.00	–	–	
Skydiver	Rate	–	1,040	–	960.00	40.00	–	–	
Loihi-MLP	Rate	99.21	150	14	99.25	660.00	128	0.76	
TrueNorth-a	Rate	92.70	1,000	28	0.27	0.27	5	0.21	
TrueNorth-b	Rate	99.42	1,000	28	108.00	108.00	30	14.06	
Tianjic	Hybrid	96.48	–	28	950.00	–	156	23.79	
This work	Temp.	98.40	26	22	0.41	15.72	42	0.04	

Enc., coding; Acc., Top-1 accuracy in percent; FPS, frames per second; CMOS technology node (Tech) in nm, power in mW, and power/neuron in uW.

Our work pushes the performance and efficiency boundary through the use of TTFS-based SNNs, and it can achieve both high accuracy and low power. We compare our results to other neuromorphic accelerators such as TrueNorth, Loihi, Systolic SNN, and Shenjing. Our work outperforms other neuromorphic accelerators in energy efficiency. This work beats Systolic SNN by 21.0× on MNIST. This work can also achieve at least 27.5× higher energy efficiency than Shenjing on MNIST. For MLP, our work is better by at least 2.4× than the state-of-the-art except for TrueNorth-b which sacrifices accuracy significantly to achieve a lower power consumption.

##### 3.4.2.3. Fashion-MNIST

With the CNN mapping algorithm proposed in this work, our work can support more complex datasets, such as Fashion-MNIST, with more complex convolutional neural networks. We compare our results to Loihi and Systolic SNN on Fashion-MNIST. As shown in Table 7, this work is able to provide improved energy efficiency over Systolic-SNN and Loihi by 49.6× and 26× on Fashion-MNIST, respectively.

**Table 7:** Comparison of this proposal with rate-based neuromorphic accelerators on the Fashion-MNIST dataset.

**Accelerator**	**Enc**.	**ACC**.	**FPS**	**Tech**	**Power (mW)**	**uJ/Frame**	**Network**
Loihi-CNN	Rate	84.30	97	14	240	2474.23	CNN
Systolic SNN-CNN-b	Rate	87.56	157	–	745	4745.22	
This work	Temp.	86.50	30	28	2.87	95.67	

##### 3.4.2.4. CIFAR10

On the CIFAR10 dataset, our work outperforms Shenjing 38.37× on energy efficiency under the same frames-per-second configuration. Although the accuracy of the TTFS algorithm on CIRAR10 dropped to 49.4%, we can increase accuracy to 87.05% by applying existing work (Stöckl and Maass, 2021) with small changes. The main difference between this work and the conventional TTFS algorithm is that this work changes neuron potentials at every end of the timesteps and fires more than one spike per neuron. Changing the neuron potential dynamically can be supported with a straight-forward modification of the spiking module in our architecture. We estimate the energy efficiency from the number of spikes of Stöckl and Maass (2021). The results show that our hardware can still achieve 3.1× better energy efficiency than Shenjing with comparable accuracy.

##### 3.4.2.5. Power and area breakdown

**Figure 7:**
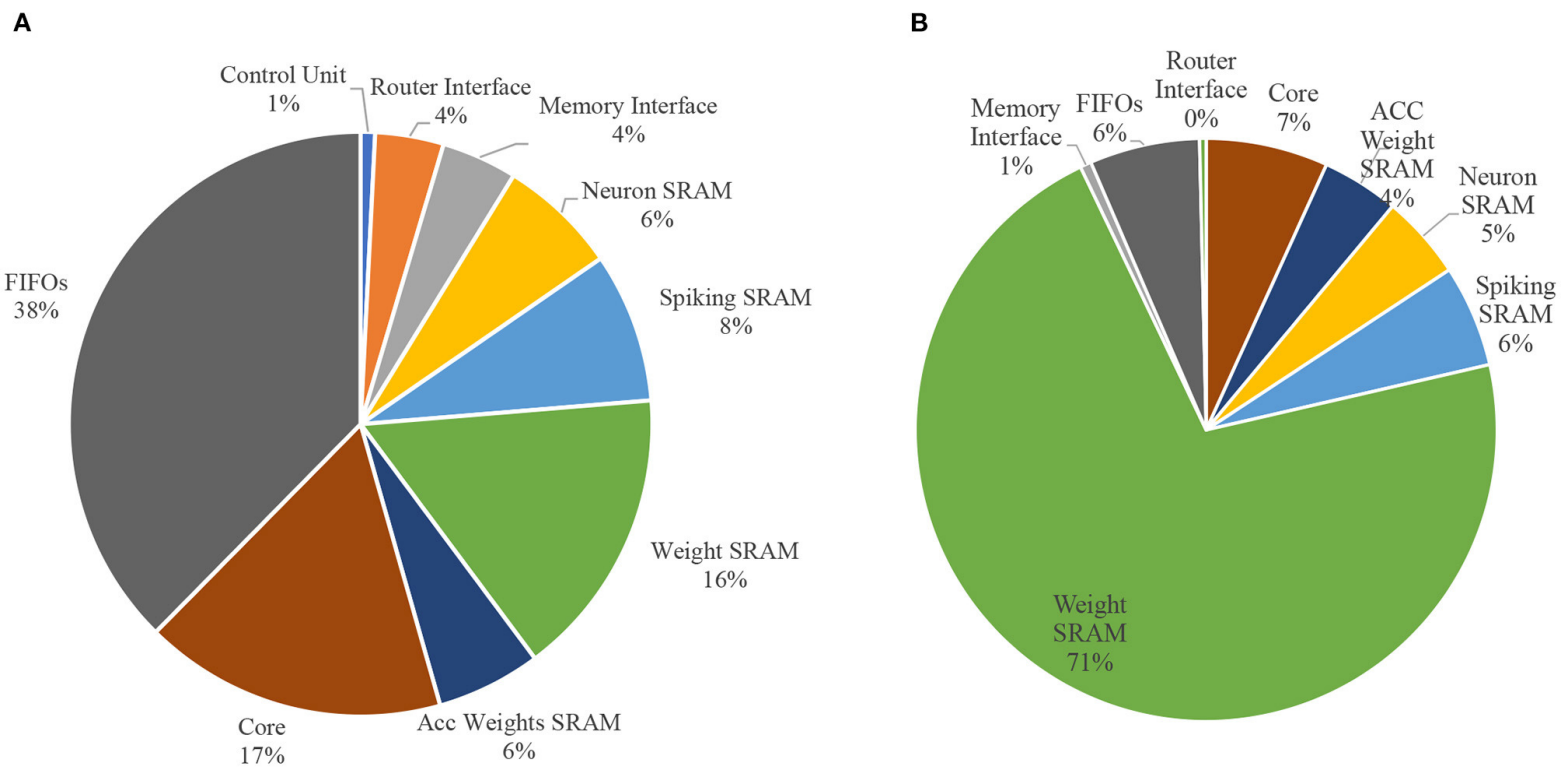
Power and area breakdown of the processing element. **(A)** Power breakdown. **(B)** Area breakdown.

Lastly, the power consumption and the area of the processing element of our accelerator are evaluated. Figure 7 provides a detailed illustration of the power and area breakdown of the hardware components inside the processing element. From the power breakdown, we find that FIFOs and SRAMs consume around 76% of the total power consumed by the processing element. From the area breakdown, the weight SRAM takes up around 71% of the total area. Using a sufficiently-sized weight SRAM, allows for higher flexibility in our design, which allows it to support models in different sizes.

#### 3.4.3. Impact of noise

**Figure 8:**
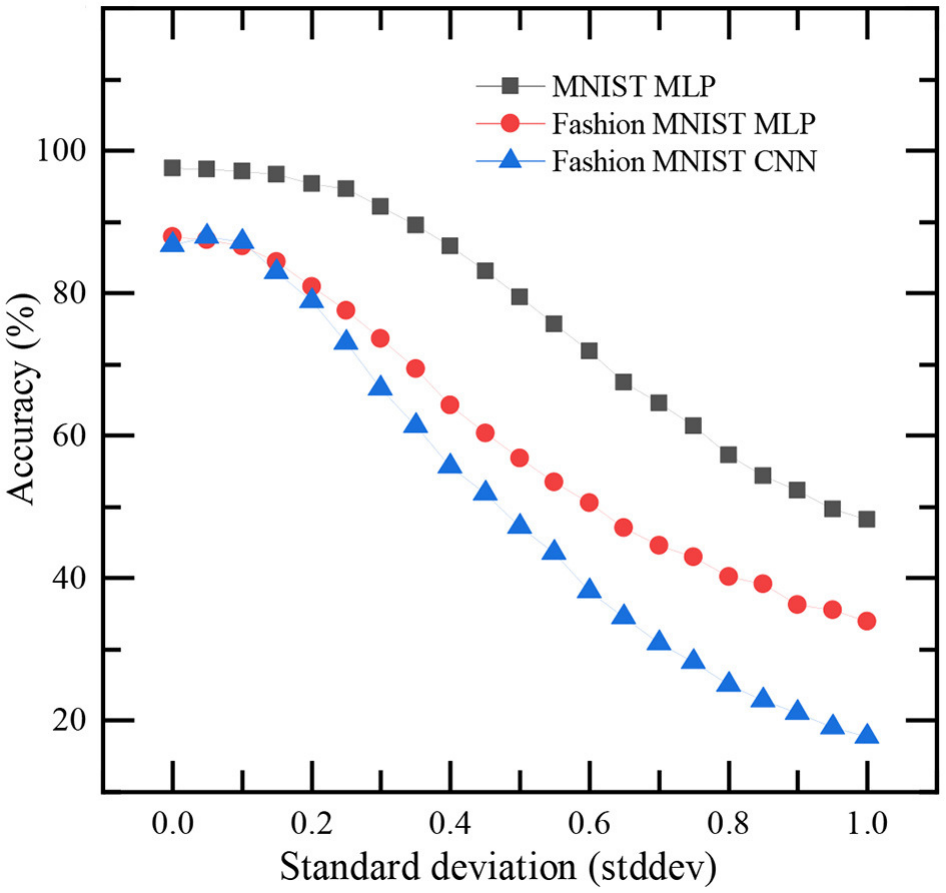
Impact of noise on the classification accuracy of this work. Gaussian noise with zero mean and a non-zero standard deviation (stddev) is added to every pixel of input images in MNIST and Fashion MNIST datasets for this study.

We present the impact of noise on the classification accuracy of TTFS-based SNNs on MNIST and Fashion MNIST datasets. After training the networks, Gaussian noise with zero mean and a non-zero standard deviation (stddev) is added to the input images to study the influence of noise on inference accuracy. The dependence of accuracy on stddev is shown in Figure 8. It can be referred from the figure that increasing the stddev beyond 0.1 significantly affects the classification accuracy.

## 4. Conclusion and discussion

In this work, we introduce our accelerator and an improved time-to-first-spike training algorithm which demonstrates the viability of temporally-encoded SNNs for image classification tasks. To address the limitations of temporally-encoded SNNs, we proposed a novel training algorithm that achieves state-of-the-art accuracy on temporally encoded SNNs. Combining this highly accurate temporal coding method with our energy-efficient hardware design improves the accuracy of TTFS-based SNNs to achieve state-of-the-art results on the MNIST and Fashion-MNIST datasets. Meanwhile, this work reduces the power consumption by at least 2.4×, 25.9×, and 38.4× over the state-of-the-art neuromorphic hardware on MNIST, Fashion-MNIST, and CIFAR10, respectively.

## Data availability statement

The original contributions presented in the study are included in the article, further inquiries can be directed to the corresponding author.

## Author contributions

MY designed hardware and algorithm, ran evaluation, and wrote the paper. TX, YT, and VM improved the idea and paper writing. BA developed the hardware platform. SP and KC developed the hardware prototype. TC guided the research and polished the paper. All authors contributed to the article and approved the submitted version.
